# The contribution of wheat to human diet and health

**DOI:** 10.1002/fes3.64

**Published:** 2015-08-14

**Authors:** Peter R. Shewry, Sandra J. Hey

**Affiliations:** ^1^ Rothamsted ResearchHarpendenHertfordshireAL5 2JQUK; ^2^ University of ReadingWhiteknightsReadingBerkshireRG6 6AHUK

**Keywords:** Diet and health, dietary fiber, grain composition, phytochemicals, wheatwheat

## Abstract

Wheat is the most important staple crop in temperate zones and is in increasing demand in countries undergoing urbanization and industrialization. In addition to being a major source of starch and energy, wheat also provides substantial amounts of a number of components which are essential or beneficial for health, notably protein, vitamins (notably B vitamins), dietary fiber, and phytochemicals. Of these, wheat is a particularly important source of dietary fiber, with bread alone providing 20% of the daily intake in the UK, and well‐established relationships between the consumption of cereal dietary fiber and reduced risk of cardio‐vascular disease, type 2 diabetes, and forms of cancer (notably colo‐rectal cancer). Wheat shows high variability in the contents and compositions of beneficial components, with some (including dietary fiber) showing high heritability. Hence, plant breeders should be able to select for enhanced health benefits in addition to increased crop yield.

## Introduction

The economic importance of wheat and its contribution to the diets of humans and livestock cannot be disputed. Currently available figures show an average annual global production of about 680 million tonnes (mt) over the 5‐year period from 2008 to 2012, with almost 700 mt being produced in 2011 (FAOStat http://faostat.fao.org/site/291/default.aspx). This makes it the third most important crop in terms of global production, the comparative values for the production of the two other major cereals over the same period being 704 mt for rice and 874 mt for maize. However, wheat is unrivaled in its range of cultivation, from 67°N in Scandinavia and Russia to 45°S in Argentina, including elevated regions in the tropics and subtropics (Feldman 1995). Furthermore, there is an increasing demand for wheat in new markets beyond its region of climatic adaptation.

Increasing global demand for wheat is based on the ability to make unique food products and the increasing consumption of these with industrialization and westernization. In particular, the unique properties of the gluten protein fraction allows the processing of wheat to produce bread, other baked goods, noodles and pasta, and a range of functional ingredients. These products may be more convenient to produce or consume than traditional foods, and form part of a “western lifestyle”.

### Wheat species

The major wheat species grown throughout the world is *Triticum aestivum*, a hexaploid species usually called “common” or “bread” wheat. However, the total world production includes about 35–40 mt of *T. turgidum* var. *durum*, a tetraploid species which is adapted to the hot dry conditions surrounding the Mediterranean Sea and similar climates in other regions. This is used for making pasta and is often referred to either as “pasta wheat” or “durum wheat”. Other wheat species are only cultivated on small areas, either for cultural reasons or for the expanding market in health foods. These are einkorn (diploid *T. monococcum* var. *monococcum)*, emmer (tetraploid *T. turgidum* var. *dicoccum*), and spelt (*T. aestivum* var. *spelta*), the latter being a cultivated form of hexaploid wheat. Spelt, emmer, and most forms of einkorn differ from bread and durum wheats in being hulled (i.e., the glumes remain tightly closed over the grain and are not removed by threshing).

### Changes in the consumption pattern of wheat

Wheat is a global commodity, with about 150 mt being traded annually (World Agricultural Outlook Board, 2014). Hence, the pattern of production does not necessarily reflect the pattern of consumption. This is particularly significant for wheat, with increased consumption being associated with the adoption of a “western lifestyle” in countries undergoing urbanization and industrialization. These include countries in which wheat is not readily grown. It is not possible to provide a global view of wheat production and consumption in the present article and we have therefore selected nine countries to illustrate trends. Data on actual consumption are not available for most countries so we use data from the FAO Food Balance Sheets (http://faostat3.fao.org/faostat-gateway/go/to/download/FB/FBS/E) on food availability *per capita*. Hence the values reflect food wasted in the home as well as food consumption.

The nine countries were selected to represent traditional wheat‐consuming areas of Western Europe (UK, Finland) and West Asia and North Africa (Turkey, Egypt), rapidly urbanizing and industrializing countries in Sub‐Saharan Africa (Nigeria, South Africa) and Asia (China, India), and Mexico in which maize and wheat are staple foods.

**Figure 1:**
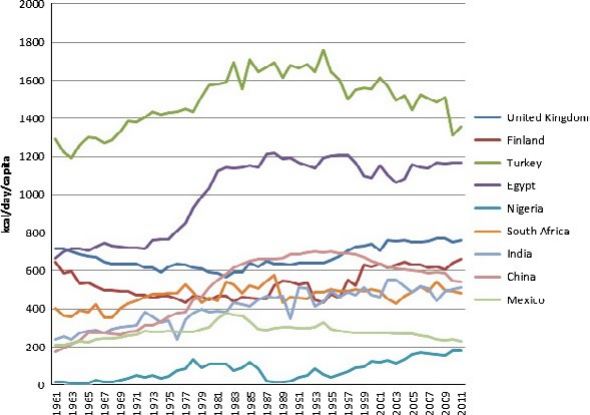
Changes in the availability of wheat (kcal/day) in nine countries between 1961 and 2011. Data from the FAO Food Balance Sheets (http://faostat3.fao.org/faostat-gateway/go/to/download/FB/FBS/E).

Figure 1 shows the changes in the availability of wheat (kcal/day) in these countries between 1961 and 2011. It is notable that the contribution of wheat to total kcal increased significantly in Nigeria (from less than 1% to 6.64%), India (11.85% to 20.41%), and China (12.20% to 17.83%), although the percentage contributions of all cereals declined in these three countries. Hence, in these three countries increased wheat consumption has occurred at the expense of other cereals, particularly minor cereals (millets and sorghum). It is also of interest to compare the figures for consumption with changes in wheat production and imports over the same period, summarized in Table 1. Whereas increased wheat production has been accompanied by decreased imports in the UK, China, and India, imports have risen dramatically in African countries, Turkey and Mexico, despite increased production.

**Table 1:** Changes in wheat production and imports in nine representative countries

	Production		Imports	
	1961–1965	2009–2013	1961–1965	2007–2011
United Kingdom	3519	13,878	4045	1178
Finland	448	872	155	24
Turkey	8584	20,844	552	3311
Egypt	1458	8472	907	9217
Nigeria	17.6	107	35	3580
South Africa	834	1813	127	1391
India	11,191	87,349	4521	606
China	19,119	118,094	5292	1912
Mexico	1783	3610	2	3358

Data are in 1000 tonnes and are 5 year averages 1961–1965 and 2009–2013 (FAOStat http://faostat.fao.org/site/291/default.aspx).

### Contribution of wheat to dietary intake of nutrients

Wheat is often considered primarily as a source of energy (carbohydrate) and it is certainly important in this respect. However, it also contains significant amounts of other important nutrients including proteins, fiber, and minor components including lipids, vitamins, minerals, and phytochemicals which may contribute to a healthy diet.

The UK National Diet and Nutrition Survey (NDNS) (Bates et al. 2014a,b) is an annual survey of the food consumption and nutritional status of a UK representative sample of 1000 people per year (500 children and 500 adults) aged 18 months and above. It is probably the most complete national survey that is available with the most recent release covering the 4 years 2008/9 to 2011/12. Data for adults (aged 19–65) are shown in Table 2.

**Table 2:** Percentage contributions of cereals and cereal products to average daily intake of some essential nutrients of adults (aged 19–64) in the UK (Bates et al. 2014a,b) (NDNS Data Released 14/05/2014) https://www.gov.uk/government/publications/national-diet-and-nutrition-survey-results-from-years-1-to-4-combined-of-the-rolling-programme-for-2008-and-2009-to-2011-and-2012)

	Total		Breads	
	Men	Women	Men	Women
Energy	31	31	12	10
Protein	23	23	11	11
Carbohydrates	45	45	21	18
NSP	40	37	21	18
Thiamin (B1)	36	35	16	15
Riboflavin (B2)	22	22	5	4
Niacin (eq) (B3)	25	26	11	10
Vitamin B6	16	18	5	4
Folates (B9)	27	27	12	12
Iron	40	38	16	15
Calcium	32	30	19	15
Magnesium	28	28	13	13
Sodium	31	31	19	18
Zinc	25	25	12	12
Copper	34	32	15	14
Selenium	28	27	13	12

NSP, nonstarch polysaccharides (dietary fiber).

Cereals and breads were the main source of energy for all age groups, contributing 31% for adults, and of nonstarch polysaccharides (dietary fiber, DF), with bread alone contributing about a fifth of the average daily intake. In addition cereals, including wheat, contribute significantly to the daily intake of protein, B vitamins, and iron. These high contributions of wheat to essential nutrients in the UK, a comparatively prosperous country with a varied diet, underline its importance to nutrition globally (not just in less developed countries).

Limited data are available for less developed countries but some comments can be made. In India, the diets of the rural poor are based predominantly on cereals, which provide 80% of energy and other nutrients except vitamins A and C. Cereals therefore require supplementation with other food groups such as pulses, vegetables, fruits, or animal products to make the diet more balanced and adequate, particularly with respect to vitamin A, iron, and riboflavin. However, Gopalan et al. (2012) suggest that the diets of the poor can also be improved to reduce the incidence of major nutrient deficiencies (such as vitamin A deficiency and iron deficiency anemia) by replacing a cereal diet (such as rice) with mixed cereals, including millets.

Changes in the Chinese diet over the past 50 years are well documented with the consumption of cereals increasing in rural communities and decreasing in urban communities between 1952 and 1992 (Du et al. 2014).

### Wheat grain composition in relation to diet and health

A vast volume of literature exists on wheat grain composition, much of which is collated in the monograph “Wheat: Chemistry and Technology”, particularly the third (Pomeranz 1988) and fourth (Khan and Shewry [Link]) editions. We will therefore focus on components of direct relevance to human nutrition and health and compare data for two fractions, wholegrain and white flour. We will also consider data on the locations of components within the different tissues of the grain, as this is relevant to their recovery in milling fractions, and briefly discuss the roles of grain components in diet and health. Some grain components, such as protein and B vitamins, have clearly established roles in the growth and health of humans and these roles will not be discussed in detail here. In other cases, such as DF and phytochemicals, the benefits are less well established and will be discussed more fully. They are also briefly summarized in Table 3.

**Table 3:** Summary of proposed and established health benefits of components present in wheat grain. A number of components have been described as having “antioxidant” properties, but the in vivo significance of this broad but readily measured activity is debated

Component	Proposed health benefit (for cereals or other foods)	Supported by approved^a^ health claim?
Dietary fiber	Reduces postprandial glycemic response (and risk of type 2 diabetes)Reduces intestinal transit timeIncreases fecal bulkReduces cholesterol and risk of coronary heart disease	Yes
	Reduces risk of colo‐rectal cancerReduces risk of breast cancerReduces risk of strokePrebiotic effectsStimulate immune responses	No
Resistant starch	Reduces postprandial glycaemic responseOther benefits as part of dietary fiber above	Yes
Fructans	Prebiotic effectsPromote calcium (and iron?) absorption	No
Betaine	Normal homocysteine metabolism (reduced risk of coronary heart disease)	Yes (not for cereals)
Choline	Normal homocysteine metabolism (reduced risk of coronary heart disease)	No
Phenolic acids	Improve vascular functionAntitumor properties“Antioxidant”	No
Alkylresorcinols	Antimicrobial“Antioxidant”	No
Lignans	PhytoestrogenAntitumorAntimicrobial	No
Sterols, stanols, and derivatives	Reduce serum cholesterol and risk of coronary heart disease	Yes
	Anticancer effects	No
Tocols	Vitamin E activity	Yes
	Prevention of neurodegenerationInduction of immune responsesAnticancerCholesterol lowering“Antioxidant”	No

a Approved by EFSA (EU) or FDA (USA).

### Protein

#### Protein content

Grain protein content is determined by genetic and environmental factors, notably the availability of nitrogen fertilization. The protein content of 12,600 lines in the USDA World Wheat Collection has been reported to range from 7% to 22% of the dry weight (Vogel et al. 1976), but generally varies from about 10–15% of the dry weight for wheat cultivars grown under field conditions. Where separate cultivars are bred for livestock and food, these may differ in protein content by about 2% protein (dw basis) when grown under the same conditions (Snape et al. 1993).

Protein is unevenly distributed in the grain, with values of 5.1% reported for the pericarp, 5.7% for the testa, 22.8% for the aleurone, and 34.1% for the germ (Jensen and Martens 1983). Broadly similar values have been reported by other authors (discussed by Pomeranz 1988b). The protein content of the starchy endosperm (white flour) is generally about 2% dry weight lower than wholegrain protein content and varies with the environment (particularly nitrogen availability), in line with the variation in wholegrain protein content.

Genetic sources of high grain protein content which have been exploited in breeding programs include Atlas 50 and Atlas 66 which are derived from the South American cultivar Frondoso (Johnson et al. 1985) and Nap Hal from India (www.indiaresource. org). Other sources of “high protein genes” have come from related wild species. For example, the Kansan variety Plainsman V contains a gene(s) from *Aegilops* which is thought to increase grain protein by 2–3% (Finney 1978). However, the most widely exploited sources of high protein genes in wheat are wild emmer (*Triticum turgidum* var *dicoccoides*) lines from Israel and in particular the accession FA15‐3 which is able to accumulate over 40% protein when given adequate nitrogen (Avivi 1978). The locus controlling this trait has been mapped to chromosome 6B and designated *Gpc‐B1* (Joppa and Cantrell 1990; Olmos et al. 2003; Distelfeld et al. 2004, 2006). Transfer of this gene into hard red spring wheats results in up to 3% more protein than in the parental lines (Khan et al.^1989^) and this effect has been exploited in the high protein hard red spring cultivar Glupro (quoted in Khan et al. 2009; Mesfin et al. 2000). The *Gpc‐B1* gene is now known to encode a transcription factor that accelerates senescence resulting in increased mobilization and transfer of nitrogen and minerals (zinc, iron) to the developing grain (Uauy et al. 2006). Hence, lines expressing this allele contain higher amounts of iron and zinc in their grain as well as higher protein (Distelfeld et al. 2006; Uauy et al. 2006).

QTLs for grain protein have also been mapped on chromosomes 5A, 5D, 2D, 2B, 6A, 6B, and 7A of bread wheat (Snape et al. 1993; Worland and Snape 2001; Blanco et al. 2002; Groos et al. 2003; Turner et al. 2004) and on chromosome 5B of emmer wheat (Gonzalez‐Hernandez et al. 2004).

#### Protein quality

Protein nutritional quality is determined by the proportions of essential amino acids, as these cannot be synthesized by animals and hence must be provided in the diet. If only one essential amino acid is limiting, the others will be broken down and excreted resulting in restricted growth in humans and loss of nitrogen present in the diet. Ten amino acids are strictly essential: lysine, isoleucine, leucine, phenylalanine, tyrosine, threonine, tryptophan, valine, histidine, and methionine. However, cysteine is often also included as it can only be synthesized from methionine, with combined proportions of cysteine and methionine often being presented. The requirements for essential amino acids are lower for adults where amino acids are required only for maintenance, than for children where they are also required for growth.

#### Essential amino acids in wheat grain

Typical contents of essential amino acids reported for wholemeal wheat and white flour are compared with the minimum physiological requirements for adults in Table 4.

**Table 4:**
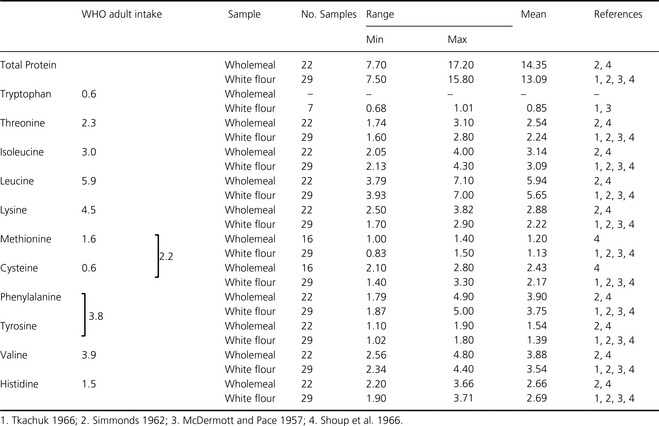
Minimum physiological requirements (g/100 g protein) for essential amino acid for adults (g/100 g protein) [WHO/FAO/UNU Expert Consultation (2002, Geneva, Switzerland)] and ranges of % total protein (%*N* × 5.7) (as is basis) and essential amino acid compositions (g/100 g protein) for wholemeal and white wheat flour

The data in Table 4 support the widely accepted view that the first limiting amino acid in wheat grain is lysine with other essential amino acids being present in adequate amounts, at least for adults. The lower contents of essential amino acids in white flour compared with wholegrain relate to the high content of lysine‐poor prolamin storage proteins (gluten proteins) in the starchy endosperm. These proteins are restricted to the starchy endosperm cells, where they account for about 80% of the total proteins, and have unusual amino acid compositions with high contents of glutamine and proline and low contents of lysine (reviewed by Shewry and Halford 2002; Shewry 2007; Shewry et al. 2009a). This contrasts with the proteins present in the other grain tissues which are more lysine‐rich.

#### Free amino acids

The pools of free amino acids in cereal grain are small (generally regarded as 5% or less of total grain nitrogen) and subjected to strict feedback regulation. Hence, analyses are rarely included when balance sheets of wheat grain composition are compiled. However, they are of interest as targets for increasing grain lysine content by transgenesis (see section below).

#### High lysine wheat

High lysine cereals have been a target for over 40 years, since Mertz et al. (1964) described the high lysine *opaque‐2* mutant of maize (reviewed by Coleman and Larkins 1999; Shewry 2007). Many other high lysine mutants were subsequently reported in maize and in other diploid cereals (sorghum and barley). However, all high lysine genes are associated with detrimental pleiotropic effects on yield and grain structure and/or composition, which have proved difficult to separate in plant breeding programs. Hence, despite a vast effort only the *opaque‐2* mutation of maize has been successfully incorporated into commercial cultivars. The molecular mechanisms responsible for *opaque‐2* and several other high lysine mutants of maize have also been determined (Schmidt et al. 1992; Coleman et al. 1995; Hartings et al. 2011). High lysine mutants have not been identified in wheat, probably due to its hexaploid constitution (dominant mutations being very rare). However, the identification of an opaque‐2‐like factor (called storage protein activator, SPA) (Albani et al. 1997) indicates that opaque‐2 like lines could be developed in transgenic wheat.

A genetic engineering approach could also be taken in wheat, as in maize where metabolic engineering has been used to increase the content of free lysine (Huang et al. 2005). However, this is unlikely to find consumer acceptance in the near future, particularly in countries where wheat is used mainly for human food rather than livestock feed.

### Carbohydrates

At maturity, the wheat grain consists of 85% (w/w) carbohydrate, 80% of which is starch (present only in the starchy endosperm); approximately 7% low molecular mass mono‐, di‐, and oligosaccharides (present in the aleurone, starchy endosperm and tissues of the embryonic axis) and fructans (present in the starchy endosperm and bran); and about 12% cell wall polysaccharides (present in all tissues) (see Stone and Morell 2009). Starch from wheat and other cereal grains is the predominant source of human dietary carbohydrate, whereas the cell wall polysaccharides are the major components of DF which is important for human health as well as having impacts on grain utilization and end‐use quality (Stone and Morell 2009).

#### Monosaccharides and oligosaccharides

The low molecular mass carbohydrate fraction includes the reducing aldohexose monosaccharide D‐glucose (0.03–0.09% of dw) and its ketohexose isomer, D‐fructose (0.06–0.08% of dw) (Lineback and Rasper 1988) and minor amounts of their phosphorylated forms, which are intermediates in carbohydrate metabolism (Stone and Morell 2009).

The most abundant oligosaccharides in wheat grain are polymers of fructose: fructo‐oligosaccharides and fructans. The distinction between these two groups is not clear so we will consider them as a single group of carbohydrates which comprise three or more fructose units, with some forms also having a single glucose unit. Cereals contain graminan‐type (branched) fructans with both *β*‐(2→1) and *β*‐(2→6) linkages (Ritsema and Smeekens 2003; Roberfroid 2005). Wholegrain fructans have been reported to have an average degree of polymerization (DP) of 5–7, with 45–50% of those in flour (Nilsson et al. 1986) and 60% of those in wholegrain (Henry and Saini 1989) having DP below 6. Studies of wheat flour have reported DP of up to 7–8 (van Loo et al. 1995) and greater than 16 (Nilsson et al. 1986).

The fructan content of whole wheat grain has been reported to vary from 0.84% to 1.85% (mean 1.28%) in 129 winter wheat varieties grown on a single site (Andersson et al. 2013), from 0.9% to 1.8% in five cultivars from five sites (Fretzdorff and Welge 2003), from 1.5% to 2.3% in 19 genotypes grown in the field (from 0.7% to 1.6% for the same lines grown in the glasshouse) (Huynh et al. 2008).

High levels of fructans are present in “sweet wheat”, a high sugar line produced by combining mutations in the granule bound starch synthase 1 (GBBS1) and starch synthase IIa (SSIIa) genes (Nakamura et al. 2006; Shimbata et al. 2011).

Analysis of milling fractions from three wheat cultivars showed the highest fructan contents in bran (3.4, 3.7, 4.0%) and shorts (3.2, 3.5, 4.1%) and less in the germ (1.7, 2.5%) and flour (1.4, 1.5, 1.7%) (Haska et al. 2008).

Wheat also contains significant amounts of the disaccharides sucrose (comprising glucose and fructose units) (0.54–1.55% of dw) and maltose (two glucose units) (0.05–0.18% of dw) and the trisaccharide raffinose (galactose, glucose, and fructose units) (0.19–0.68% of dw) (Lineback and Rasper 1988). Huynh et al. (2008) reported from 0.39% to 0.49% raffinose in 19 genotypes grown in the field, and from 0.15% to 0.30% in the same lines grown in the glasshouse.

##### Health benefits of fructans

Fructans form part of the DF fraction and are highly fermentable in the colon, with inulin (a form of fructan derived from Jerusalem artichoke and other plants) being widely used as a standard prebiotic (discussed below under dietary fiber).

However, in wheat, fructans and raffinose are the major components of a group of small fermentable carbohydrates which have been termed FODMAPs (Fermentable, Oligo‐, Di‐, Mono‐saccharides, and Polyols). It has been suggested that a low FODMAP diet improves the management of irritable bowel syndrome (IBS) and inflammatory bowel disease (Crohn's disease and ulcerative colitis), by reducing fermentation in the colon (Gibson and Shepherd 2010; Muir and Gibson 2013). Hence, low fructan wheats could be of interest for developing low FODMAP food products.

Fructans have received particular attention in relation to human health because of their reported ability to promote the absorption of calcium (Lopez et al. 2000; Scholz‐Ahrens et al. 2001; Abrams et al. 2007a,b). They have also been suggested to increase iron absorption, but the evidence for this is less conclusive (see, for example, Ohta et al. 1998; Patterson et al. 2009). Of particular interest is a recent report that wheat lines differing in arabinoxylan and fructan content had no effect on iron status when fed to iron deficient broiler chickens (Tako et al. 2014).

#### Starch

Starch constitutes about 60–70% of the mass of wheat grain, and about 20% more of the total mass (i.e., about 70–85%) in white flour (Toepfer et al. 1972). It influences aspects of end‐product quality and is crucial for human nutrition, being the main source of dietary carbohydrate.

Starch is a mixture of two glucose polymers: amylose, which comprises single unbranched (1→4) *α*‐linked chains of up to several thousand glucose units and amylopectin which is highly branched (with (1→6) *α*‐linkages as well as (1→4) *α*‐linkages) and may comprise over 100,000 glucose unit residues. In most species, including wheat, amylose and amylopectin occur in a ratio of 1:3 amylose: amylopectin.

##### Resistant starch

Most starch is digested in the small intestine but a proportion may escape digestion and is termed “resistant starch” (RS) (Englyst and Cummings 1985; Asp 1992). RS is classified into five types, with starch entrapped in the food matrix and therefore physically inaccessible being termed RS1, native (uncooked) starch granules RS2, retrograded starch formed after starch gelatinization RS3, chemically modified starch RS4 and starch capable of forming complexes between amylose and long branch chains of amylopectin with lipids RS5 (Thompson 2000; Sharma et al. 2008; Hasjim et al. 2010; Birt et al. 2013). In fact, the resistance of starch to digestion is influenced by many properties of the starch granule including size, shape, and crystallinity, and the contents of amylose, lipids, proteins, and phosphate (Themeier et al. 2005; Tester et al. 2006) and also depends on the processing conditions (Alsaffar 2011).

The RS content of food is highly variable, with cereals generally containing about 3% RS and green bananas about 75%. RS forms part of DF and has similar properties to nonstarch polysaccharides (NSP) in that it is fermented by colonic microorganisms into short chain fatty acids (SCFAs) (acetate, butyrate, and propionate). However, it is associated with the production of higher levels of butyrate compared to other types of DF (Topping and Clifton 2001; Topping et al. 2008) and hence may have greater health benefits. Similarly to NSP, RS increases fecal bulk, by increasing the volume of bacteria, and may also have beneficial effects on insulin sensitivity (Robertson et al. 2003, 2005; Lobley et al. 2013) and reduce the risk of colo‐rectal cancer (Keenan et al. 2012; Humphreys et al. 2014). However, RS is a minor component compared with NSPs in western diets (Lobley et al. 2013). For example, the daily intake of RS in European countries is estimated as 4.1 g/day RS compared with 15–20 g/day of fermentable fiber (Cummings 1983). Hence, the beneficial effects of RS in western diets are minor in comparison with those of NSPs in bran, fruit, and vegetables (Cummings et al. 2004).

The demonstration that the amount of RS2 and the ability to form RS3 are influenced by the amylose content of cereal starches (Morell et al. 2004; Rahman et al. 2007) has resulted in interest in identifying or developing lines with increased amylose content.

#### Mutations affecting starch composition

Naturally occurring and induced mutations affecting the ratio of amylose: amylopectin were identified many years ago in diploid cereals (maize, barley, rice) but the polyploidy nature of wheat limited their identification and exploitation in wheat until the last 25 years.

##### Waxy wheat

Low amylose (waxy) cereals have essentially 100% amylopectin and result from mutations in granule bound starch synthase (GBSS), the single enzyme that catalyses amylose synthesis. Waxy cereal starches have unusual properties (notably high viscosity and water‐retention) (Kim et al. 2003) that are exploited in the food industry, particularly for the production of refrigerated and frozen foods and as fat replacers (Jobling 2004), whereas waxy (glutinous/sticky) rice is widely consumed in Asia. In polyploid bread wheat, the waxy phenotype requires mutations in all three GBSS genes and has not been reported to occur naturally. However, the inactivation of one or two of these genes results in the production of partial waxy lines which occur quite commonly. For example, Yamamori et al. (1994) screened 1960 wheat cultivars and identified null mutations at all three loci, although these differed in frequency and geographical distribution: Wx‐1A nulls occurred in 16.2% of Japanese, 10.8% of Korean, and 51.9% of Turkish wheats but were less common in Chinese, South Asian, Australian, North American, Western European, and Russian wheats, whereas Wx‐1B nulls were commonest in Australian and Indian wheats. Only one Wx‐1D null was identified (in a Chinese cultivar), whereas nine Japanese cultivars were double Wx‐1A/Wx‐1B nulls. Demeke et al. (1997) similarly reported high incidences of Wx‐1A and Wx1‐B nulls in Japanese and US wheats, respectively; while Graybosch et al. (1998) reported 10% of a sample of 200 US wheats had null Wx alleles: six Wx‐1A nulls, 13 Wx‐1B nulls and one double Wx‐1‐A/Wx‐1B null. These partial waxy wheats have lower amylose contents than normal wheats, with the Wx‐1B null having a greater effect than the Wx‐1A null, and the double null a greater effect than the single nulls (Yamamori et al. 1994; Graybosch 1998; Graybosch et al. 1998).

Partial null lines can be readily crossed to obtain complete waxy types of bread and durum wheat with very low amylose contents (about 0–2%) (Nakamura et al. 1995; Kiribuchi‐Otobe et al. 1997; Graybosch 1998; Lafiandra et al. 2010). Polymorphism at the waxy loci has also been shown to affect the amount of amylose (increased or decreased) in durum and bread wheats (Yamamori 2009).

In contrast to high amylose starch (below), waxy starch is highly digestible and hence has a high glycemic index.

##### High amylose wheat

Analysis of diploid cereals showed that the high amylose phenotype could arise from reduced activity of starch synthase (SSII) or starch branching enzymes (SBEIIa or SBEIIb) (reviewed by Lafiandra et al. 2014). As with waxy wheat, mutations in all three genomes are required to significantly affect the amylose content and naturally occurring high amylose lines have not been reported. Yamamori and Endo (1996) therefore used biochemical screening to identify mutants lacking each of the three SSIIa proteins. Combining these three mutations resulted in lines with about 20% less starch but amylose contents of 37% (Yamamori et al. 2000). Similarly, Rakszegi et al. (2014) recently reported amylose contents of about 40% when the three mutations were introgressed into three commercial bread wheat cultivars, whereas Lafiandra et al. (2010) have used the mutations on the A and B genomes to produce a SSIIa *null* line of the durum wheat cultivar Svevo with an amylose content of 43.6% (compared to 23% in the control).

Hogg et al. (2013) identified two A genome SSIIa nulls but no B genome nulls from a screen of 255 *T. durum* accessions. These were crossed into a commercial cultivar and mutated to generate double SSIIa nulls with 44.3% and 42.8% amylose compared to 28.7% in the control.

The development of TILLING technology for wheat (Slade et al. 2005) has facilitated the identification of further mutations in genes of starch synthesis (Uauy et al. 2009; Sestili et al. 2010). Thus, Botticella et al. (2011) identified knock‐out mutants in the three *SBEIIa* homeologues of bread wheat and showed that combining two of these resulted in increases in amylose content from 33.2% in the control to between 38.6% and 39.9%, whereas Hazard et al. (2012) showed that double *SBEIIa* mutants in durum wheat had an increase of 22% in amylose content and 115% in resistant starch content. Similarly, Slade et al. (2012) combined mutations in *SBEIIa* genes to produce durum and bread wheat lines containing 47–55% amylose and with elevated levels of resistant starch compared with wild‐type wheat.

However, high amylose wheats differ in their processing properties from conventional wheats, particularly in starch swelling and viscosity (Van Hung et al. 2005; Yamamori et al. 2006; Schirmer et al. 2013), which means that processes and products will need to be modified.

##### Yields of starch mutants

Because commercial waxy and high amylose cultivars of wheat have not been developed data on comparable yields are not readily available. However, it is expected that both phenotypes would be associated with yield deficits, as reported for commercial high amylose and waxy maize hybrids. This is currently about 3–4% for waxy maize but greater for high amylose lines. Similarly, transgenic high amylose barley lines were shown to have a yield deficit of over 20% (Carciofi et al. 2012). Effects on yield are not unexpected, as starch is the major determinant of yield and any changes in composition are likely to affect the highly organized packaging of the amylose and amylopectin polymers in the starch granules.

Nevertheless, it should be possible to produce lines with improved health benefits, and acceptable yields and processing properties, by increasing the amylose content by a modest amount (e.g., by using mutations in only one or two of the genomes).

### Cell wall polysaccharides

The major components of wheat grain DF are cell wall polysaccharides, lignin, fructan, and resistant starch (see above and Table 5).

**Table 5:** Contents of total dietary fiber and dietary fiber components in 129 winter wheat varieties (taken from data in Andersson et al. 2013)

	Range	Mean
Total dietary fiber (%)	11.5–15.5	13.4
Klason lignin (%)	0.74–2.03	1.33
Arabinoxylan (%)	5.53–7.42	6.49
Cellulose (%)	1.67–3.05	2.11
*β*‐Glucan (%)	0.51–0.96	0.73
Fructan (%)	0.84–1.85	1.28

Lignin is a complex polymer of aromatic alcohols and is characteristic of the secondary cell walls of woody tissues. In wheat and other cereals it is only present in the pericarp/seed coat (Stone and Morell 2009) and hence is enriched in the bran and absent from white flour (Table 6). The detailed structure of cereal grain lignin has been described by Bunzel et al. (2004).

**Table 6:** Contents and compositions of cell wall in wheat grain tissues (% dw) (from Shewry et al. 2010)

Tissue	Cell walls (% dw)	Components				
		Cellulose	Lignin	Xylan	*β*‐glucan	Glucomannan
Starchy endosperm	2–3	2	0	70	20	7
Bran		29	8	64	6	–
Aleurone	40	2–4	0	62–65	29–34	–
Outer pericarp (beeswing)		30	12	60	–	–

The major cell wall polysaccharides of wheat grain are arabinoxylan (AX) and (1→3, 1→4)‐*β*‐D‐glucan (*β*‐glucan), with smaller amounts of cellulose ((1→4)‐*β*‐D‐glucan) and glucomannan (Table 5). Other minor polysaccharides, including callose ((1→3)‐*β*‐D‐glucan), xyloglucan, and pectins, can be detected by immunocytochemistry (Pellny et al. 2012; Chateigner‐Boutin et al. 2014; Palmer et al, 1945) or sugar analysis.

AX comprises a backbone of *β*‐D‐xylopyranosyl (xylose) residues linked through (1→4) glycosidic linkages with some residues being substituted with *α*‐L‐arabinofuranosyl (arabinose) residues at either one or two positions. Some arabinose residues present as single substitutions on xylose may also be substituted with ferulic acid at the five position, allowing the oxidation of ferulate present on adjacent AX chains to give dehydrodimers (diferulate cross‐links). The extent of diferulate cross‐linking is important as it affects the physio‐chemical properties (notably solubility and viscosity) of AX and hence the behavior in food processing and also probably the health benefits. AX is therefore often divided into two classes, depending on whether it is extractable (WE‐AX) or unextractable (WU‐AX) with water.

*β*‐glucan comprises glucose residues joined by (1→3) and (1→4) linkages. Single (1→3) linkages are usually separated by two or three (1→4) linkages, but longer stretches of up to 14 (1→4) linked glucan units (sometimes referred to as “cellulose‐like” regions) have been reported for wheat bran *β*‐glucan (Li et al. 2006). Unlike *β*‐glucan in oats and barley, wheat *β*‐glucan shows low solubility, with about 10–15% of the total in wholemeal samples being soluble in hot water (Nemeth et al. 2010).

The amounts and proportions of cell wall polysaccharides vary between tissues, as summarized in Table 6.

The cell walls of the starchy endosperm (i.e., white flour) account for about 2–3% of the dry weight and comprise about 70% AX and 20% *β*‐glucan, with 2% cellulose and 7% glucomannan (Mares and Stone 1973). In addition, immunolabeling of developing tissues has shown the presence of callose ((1→3)‐*β*‐D‐glucan), xyloglucan and pectin (Pellny et al. 2012; Chateigner‐Boutin et al. 2014; Palmer et al. 2015).

Starchy endosperm AX contains only low levels of ferulic acid: 0.2–0.4% (w/w) of WE‐AX and 0.6–0.9% (w/w) of WU‐AX (Bonnin et al. 1998).

The aleurone cells have thick cell walls accounting for about 35–40% of the dry weight (Barron et al. 2007). These comprise 29% *β*‐glucan, 65% arabinoxylan and 2% each of cellulose and glucomannan (Bacic and Stone 1981). The aleurone AX are highly esterified and cross‐linked with about 3.2% of the AX dw being ferulic acid and 0.45% being diferulic acid (Antoine et al. 2003; Parker et al. 2005). Additional esterification with *p*‐coumaric acid and acetyl groups also occurs (Rhodes and Stone 2002; Antoine et al. 2004).

The outer layers comprise about 45–50% cell wall material (Barron et al. 2007). The major pericarp tissue comprises about 30% cellulose, 60% arabinoxylan, and 12% lignin (reviewed by Stone and Morell 2009). The pericarp AX also has a complex highly branched structure, with galactose and glucuronic acid residues, and is often termed glucuronoarabinoxylan (GAX). It also has high contents of ferulic acid and diferulic acid (Saulnier and Thibault 1999; Antoine et al. 2003; Parker et al. 2005) and acetylation (Mandalari et al. 2005) with significant amounts of ferulic acid trimer (Barron et al. 2007). Immunolabeling shows that the aleurone and pericarp cell walls also contain pectic polysaccharides (Chateigner‐Boutin et al. 2014; Palmer et al. 2015).

Barron et al. (2007) reported that the scutellum and embryonic axis of the germ contained about 12% and 25% of neutral carbohydrate, respectively, with arabinose and xylose (presumably derived from AX) accounting for about 65% of the total. Other sugars released were glucose (presumably from *β*‐glucan), galactose and, for the embryonic axis only, mannose (possibly from mannans or glucomannans).

##### Variation in cell wall polysaccharides in wholegrain and white flour

Table 7 summarizes analyses of total dietary fiber (TDF) and the major components (AX and *β*‐glucan) in wholemeal and white flour, with the AX being determined as total, water‐extractable (i.e., soluble) and water‐unextractable (insoluble). Substantial variation occurs in all the total contents of all fractions, with wholegrain being richer in TDF and individual DF components than white flour.

**Table 7:** Ranges of cell wall polysaccharides in wholegrain wheat and white flour (summarized from Shewry 2013)

Components (g/100 g dw)	Sample	No. samples	Range		Mean	References
			Min	Max		
Total Dietary Fiber	Wholemeal	138	10.26	15.5	13.39	1, 2, 3, 4, 5
	White flour	10	1.94	6.27	3.52	2, 4, 5, 6
Total AX	Wholemeal	173	5.53	8.88	6.60	1, 7, 8, 9
	White flour	110	1.88	3.58	2.64	9
WE‐AX	Wholemeal	166	0.29	1.62	0.57	10
	White flour	110	0.30	0.91	0.58	9
WU‐AX	Wholemeal	20	5.87	8.16	6.61	8
	White flour	90	1.52	2.93	2.10	9
*β*‐glucan	Wholemeal	166	0.29	1.10	0.81	10
	White flour	–	–	–	–	–

1. Andersson et al. 2013; 2. USDA National Nutrient Database R26; 3. UK Cofids database; 4. Finland Finelli database; 5. South African Food Composition database; 6. Turkey Türkomp Food Composition database; 7. Barron et al. 2007; 8. Saulnier et al. 1995; 9. Saulnier et al. 2007; 10. Gebruers et al. 2008.

Of particular interest are the datasets from the EU HEALTHGRAIN study which analyzed lines grown together on a single site (Gebruers et al. 2008, 2010; Andersson et al. 2013). Hence the data are directly comparable. In this study TOT‐AX in white flour varied by over twofold (from 1.35 to 2.75% dw), and WE‐AX by over fourfold (0.30–1.40% dw). The proportion of soluble AX ranged from 20% to 50% of TOT‐AX.

#### Health benefits of dietary fiber

There is a massive volume of literature supporting the health benefits of wheat fiber, although a wide range of fractions have been studied. The UK Scientific Advisory Committee on Nutrition (SACN) (2015) has recently reviewed the evidence for health benefits of cereal fiber, as part of a wider review of dietary carbohydrates. Table 8 therefore summarize its conclusions on the health benefits of wheat fiber, including data for “total fiber”, “soluble fiber” and “insoluble fiber” fractions which include fiber from other sources (fruit, vegetables, legumes). These tables also include data for widely studied *β*‐glucan fractions from oats and barley. However, it should be noted that oat and barley *β*‐glucans differ from wheat *β*‐glucan in their structures and properties, being much more soluble and giving more highly viscous solutions (Li et al. 2006; Lazaridou and Biliaderis 2007).

**Table 8:** Summary of the conclusions of SACN 2015 on associations and effects (in italics) of cereal and other dietary fiber fractions on improved cardio‐metabolic and colo‐rectal health

Health outcome	Fraction
Cardio‐metabolic health	
CVD	TDF, insoluble fiber, soluble fiber, total cereals, wholegrains
Coronary events	TDF, insoluble fiber, cereal fiber, high fiber breakfast cereals
Stroke	TDF, wholegrains
Hypertension	Wholegrains
Blood pressure	*Oat bran/oat or barley β‐glucans*
Fasting blood total cholesterol, LDL cholesterol, triacylglycerol	*Oat bran/oat or barley β‐glucans*
Type 2 diabetes	TDF, insoluble fiber, soluble fiber, cereal fiber, high fiber breakfast cereals, whole grain bread, wholegrains
Colo‐rectal health	
Fecal weight	*TDF, wheat fiber, non‐wheat cereal fiber*
Intestinal transit time	*TDF, wheat fiber, non‐wheat cereal fiber*
Intestinal transit time in patients with constipation	*Wheat fiber*
Constipation	*Cereal fiber*
Colo‐rectal cancer	TDF, cereal fiber
Colon cancer	TDF, cereal fiber, wholegrains
Rectal cancer	TDF, wholegrains

SACN 2015 is the most comprehensive and critical review so far published but even this did not cover the full literature: the authors noted that: “due to the wealth of data available and because of the concerns around their limitations, case‐control, cross‐sectional and ecological studies were not considered” with “only prospective cohort studies and randomized controlled trials” being considered.

It is therefore important to mention other studies which did not meet the SACN criteria but are nevertheless relevant to this review. In particular, several studies have reported decreased risk of other types of cancer. For example, cereal fiber and breast cancer in premenopausal women (Cade et al. 2007), fiber/wholegrain intake and small intestinal cancer in men and women (Schatzkin et al. 2008) and wholegrains/high fiber foods and pancreatic cancer (Chan et al. 2007).

In most countries claims for health benefits of foods and food ingredients must be approved by regulatory authorities, which are the FDA in the USA and EFSA in the EU. These bodies require high standards of evidence with a relatively small proportion of applications being approved. Nevertheless, 11 out of about 250 health claims approved by EFSA relate to wheat and related cereals (barley, oats, rye) (http://ec.europa.eu/nuhclaims/), and in particular to fiber components and their effects on GI tract function (reduced transit time, increased fecal bulk), reduction in postprandial glucose responses, or maintenance of blood cholesterol concentrations. These claims can be regarded as generally accepted.

#### Mechanism of action of dietary fiber

A full discussion of the mechanisms of action of DF is outside the scope of this review, except to note that a number of mechanisms probably contribute, including physical properties (fecal bulk, viscosity of soluble fiber fractions) and fermentation in the colon to produce SCFAs which have physiological effects on the colon and other tissues. A number of reviews of this topic are available (Topping 2007; Buttriss and Stokes 2008; Theuwissen and Mensink 2008; Anderson et al. 2009; Brownlee 2011; Lafiandra et al. 2014).

The concept of stimulating the growth of beneficial colonic bacteria by manipulating the content and composition of nondigestible carbohydrates in the diet (prebiotics) was introduced by Gibson and Roberfroid (1995), following the introduction of microbial (probiotic) supplements. Although these two approaches have the same aim and are essentially complementary, it is considered that the effects of prebiotics may be less transitory than those of probiotics. The effects of prebiotics may be determined experimentally in culture, using either sophisticated model colon systems or simple cultures of fecal bacteria, with effects on bacterial populations (in particular increases in *Bifidobacterium* and *Lactobacillus* spp.) and the production of SCFAs (particularly butyrate) being measured.

The most widely studied prebiotic (which is usually used as a standard) is inulin, a *β*‐(2→1) linked fructan from tubers of Jerusalem artichoke (*Helianthus tuberosus)*. Fructans are components of the DF fraction in wheat (see above) and other DF components (resistant starch and all nonstarch polysaccharides) are similarly fermented, although the extent depends on their solubility and other factors such as lignification, particle size and effects of food processing. In an excellent review, Cummings and Macfarlane (1991) estimate that at least 50% of the cellulose and 80% of the noncellulosic polysaccharides in the human diet are fermented in the colon.

There are also differences in the proportions of individual SCFAs produced by fermentation of different oligosaccharides and polysaccharide substrates. Although the precise ratios of SCFAs produced by fermentation in vitro vary between reports, two parallel studies using in vitro fecal cultures showed that wheat arabinoxylan fractions gave broadly similar proportions of SCFAs to inulin whereas five *β*‐glucan fractions from oats and barley gave substantially higher proportions of butyrate than inulin (Hughes et al. 2007, 2008). Hence, fructans and other prebiotic oligosaccharides (including the arabinoxylan oligosaccharides (AXOS) which have approved health benefits) should be considered as highly fermentable DF components.

#### Dietary fiber and immune function

There is increasing evidence that mixed‐linkage *β*‐glucans are able to regulate the immune responses that are involved in fighting infection, attacking tumors and various inflammatory conditions. Most studies have been carried out on (1→3) (1→6) *β*–glucans from yeast, mushrooms, other fungi and seaweed. Cereal *β* ‐glucans differ for these species in their linkage pattern (with (1→3) (1→4) as opposed to (1→3) (1→6) linkages) but may also have immune modulatory properties (Brown and Gordon 2001; Rice et al. 2005). Immune stimulatory effects have also been proposed for arabinoxylan (Capek and Matulova 2013; Li et al. 2015).

These effects remain to be conclusively demonstrated in vivo and the mechanisms at the organism, as opposed to cellular, level remain unclear. However, they could prove to be an important facet of the contribution of wheat to human health.

### Phytochemicals: phenolics and terpenoids

Wheat grain contains two major groups of phytochemicals derived from different biosynthetic pathways: phenolics and terpenoids. A range of health benefits have been proposed for these components but few have been established with sufficient scientific rigor to result in health claims approved by FDA or EFSA, the main exceptions being tocols (vitamin E) which have a number of established functions and plant sterols and stanol esters which have accepted benefits in reducing blood cholesterol and therefore the risk of cardiovascular disease.

#### Phenolic compounds

These contain at least one aromatic ring bearing at least one hydroxyl group. They exhibit immense diversity in structure, forming the largest and most complex group of secondary products present in cereal grain.

Phenolic acids (PAs) are the major group of phytochemicals in wheat grain. They contain a phenolic ring and an organic carboxylic function and fall into two groups, which are derived either from cinnamic acid or benzoic acid. They also occur in three forms, either as free compounds, as soluble conjugates bound to low molecular weight compounds such as sugars, and as bound forms which are linked to cell wall polysaccharides (particularly arabinoxylan in cereal grain) by ester bonds.

Alkylresorcinols are phenolic lipids comprising a 1,3‐dihydroxylated benzene ring with an alkane chain at position 5.

Flavonoids are a large and structurally highly diverse group of phenolic compounds which are based on a 15 carbon ring structure (Jende‐Strid 1993). Over 5000 have been characterized from plants and classified into a number of groups. Of particular importance and interest in wheat are the flavanols (flavan‐3‐ols), which are based on a 2‐phenyl‐3,4‐dihydro‐2H‐chromen‐3‐ol skeleton and include the proanthcyanidin pigments which are present in the testa of red wheats, and the anthocyanidins which are glycosylated to form anthocyanins.

Lignans are polyphenols derived from phenylalanine via dimerization of substituted cinnamic alcohols, to a dibenzylbutane skeleton.

#### Terpenoids

Terpenoids are derived from five carbon isoprene units.

Plant sterols are steroid alcohols, comprising a tetracyclic cyclopenta[*α*]phenanthrene ring with a hydroxyl group at the C4 position and a flexible side chain at the C17 carbon position. They are divided into three types, the 4‐desmethyl sterols which are the major components in plant tissues and the minor 4*α*‐monomethyl sterols and 4,4‐dimethyl sterols which are precursors of the 4‐desmethyl sterols. The major plant 4‐desmethyl sterols have a Δ^5^ double bond in the B ring and modifications at the C24 position in the side chain (Piironen et al. 2000). Cereals also contain significant amounts of saturated sterols, which are called stanols. A substantial proportion of the sterols and stanols present in wheat are modified, with the 3OH group on ring A being esterified to a fatty acid or phenolic acid to form sterol esters, or *β*‐linked to a carbohydrate to form a sterol glycoside, with the latter also sometimes being acylated. *β*‐sitosterol (the major wheat sterol) is present in approximately equal amounts in all four forms (free, esterified, glycosides, and acylated glycosides) (Chung et al. 2009). However, the modified forms are usually hydrolyzed during preparation to release the free sterols. Unless otherwise noted all data presented here are for total sterols (after hydrolysis).

Tocols comprise a chromanol ring with a C16 phytol side chain, which can be either saturated (tocopherols, T) or contain three double bonds at carbons 3, 7, and 11 (tocotrienols, T‐3). Each type also exists in four forms, which differ in the positions of methyl groups on the chromanol ring and are called *α* (5,7,8‐trimethyl), *β* (5,8‐dimethyl), *γ* (7,8‐dimethyl), and *δ* (8‐methyl).

Carotenoids are isoprenoids derived from long polyene chains of 35–40 carbons. They occur in two major forms, the oxygen‐containing xanthophylls (which include lutein and zeaxanthin) and the unoxygenated carotenes (which include *α*‐carotene and *β*‐carotene). Some carotenoids, including the carotenes, are converted to vitamin A (retinol) in mammals, and hence are also referred to as provitamin A. The levels of carotenoids in cereal grain are generally low, but are of particular interest in durum wheat where lutein in the major determinant of the yellow color which is a quality trait for breeders and consumers (Borrelli et al. 2000).

#### Content and composition of phytochemicals in wholegrain and flour

Although many studies of phytochemicals in wheat have been published, very few provide comparative analyses of multiple genotypes which have been grown under the same conditions and analyzed using the same methods. Hence, much of the discussion below is based on the outcomes of the HEALTHGRAIN study which compared wholemeal samples of 150 wheat genotypes grown together on a site in Hungary (Ward et al. 2008), with 26 lines then being grown in five additional environments (Shewry et al. 2010). This material was analyzed for phytochemicals and other putative “bioactive” components and provides the most complete database on wheat grain composition so far reported. Most other studies have also been carried out on wholemeal with limited data on white flour being available.

Table 9 therefore summarizes wholegrain data from the HEALTHGRAIN study and from the most extensive reported studies for carotenoids (Moore et al. 2005) and lignans (Smeds et al. 2009), with data on total phenolic acids in two samples of white flour. The reader is referred to Piironen et al. (2009) and Chung et al. (2009) for detailed recent reviews of phenolics and terpenoids in wheat.

**Table 9:** Variation in the contents of phytochemicals (phenolics and terpenoids), in wholegrain (WG) of wheat and white flour

Component	Fraction	Lines	Range	Fold variation	Mean	References
Phenolics						
Total phenolic acids	WG	150	326–1171 *μ*g/g dm	3.4	657 *μ*g/g dm	1
Total phenolic acids	White flour	2	171–190 *μ*g/g dm	1.1	180 *μ*g/g dm	2
Free phenolic acids	WG	150	3–30 *μ*g/g dm	10	10.6 *μ*g/g dm	1
Conjugated phenolic acids	WG	150	76–297 *μ*g/g dm	3.9	162.5 *μ*g/g dm	1
Bound phenolic acids	WG	150	208–878 *μ*g/g dm	4.2	484.9 *μ*g/g dm	1
Bound ferulic acid	WG	150	162–721 *μ*g/g dm	4.5	367.4 *μ*g/g dm	1
Alkylresorcinols	WG	150	241–677 *μ*g/g dm	2.8	432 *μ*g/g dm	1
Lignans	WG	73	3.4–22.70 *μ*g/g db	6.7	10.5 *μ*g/g db	3
Terpenoids						
Total tocols	WG	150	27.6–79.7 *μ*g/g dm	2.9	49.9 *μ*g/g dm	4
Tocopherols	WG	150	12.3–33.2 *μ*g/g dm	2.7	19.9 *μ*g/g dm	4
*α*‐Tocopherol (vitamin E)	WG	150	9.1–19.9 *μ*g/g dm	2.2	13.6 *μ*g/g dm	4
Tocotrienols	WG	150	12.5–52.0 *μ*g/g dm	4.2	30.02 *μ*g/g dm	4
Total sterols (inc stanols)	WG	150	670–959 *μ*g/g dm	1.43	844 *μ*g/g dm	5
% stanols	WG	150	11–29%	2.6	23.9%	5
*β*‐carotene	WG	8	0.11–0.24 *μ*g/g db	2.1	0.19 *μ*g/g db	6
Lutein	WG	8	0.93–1.3 *μ*g/g db	1.4	1.14 *μ*g/g db	6
Zeaxanthin	WG	8	0.23–0.44 *μ*g/g db	1.9	0.33 *μ*g/g db	6

Where original data were presented on an “as is” basis, a moisture content of 14% was assumed to convert to dry matter basis (db).

References: 1. Li et al. 2008; 2. Mattila et al. 2005; 3. Smeds et al. 2009; 4. Lampi et al. 2008; 5. Nurmi et al. 2008; 6. Moore et al. 2005. Stanols are expressed as % total sterols+stanols.

PAs are the major group, ranging in amount from about 300 to 1500 *μ*g/g dry weight. Bound PAs account for about 70–80% of the total PAs, with ferulic acid being the major bound component (and hence the major component of total PAs). Ferulic acid is known to be bound to arabinoxylan by ester linkages to the arabinose side chains. This allows the formation of cross‐links between arabinoxylan chains, by oxidation to give diferulates (dehydrodimers) or more rarely triferulate. The amounts and compositions of PAs vary immensely between cultivars, with free PAs being particularly variable (Li et al. 2008). Alkylresorcinols and lignans also show wide variation, from 241 to 677 *μ*g/g dry weight and from 3.4 to 22.7 *μ*g/g dry weight, respectively (Table 9).

Wide variation also occurs in the contents of terpenoids, with data for the major fractions and *α*‐tocopherol determined in large scale studies being given in Table 9. It is notable that the content of total sterols (including stanols) varied less than those of other phytochemicals (by only ×1.43), possibly because sterols play essential functions as components of plant cell membranes. There was also variation in the percentage of stanols, by 2.6‐fold (from 11 to 29% total sterols).

Carotenoids are minor components in bread wheat with few detailed studies. The “major” component in wholegrain is lutein, with lower amounts of *β*‐carotene and zeaxanthin. These vary by up to twofold between cultivars (Table 9).

#### Distribution of phytochemicals in mature grain

All phytochemicals are concentrated in the aleurone and bran (embryo and outer layer), with data for white flour fractions rarely being reported. For example, the two white flour samples analyzed for PAs by Mattila et al. (2005) contained 171 and 190 *μ*g/g dm compared with 326–1171 *μ*g/g dm in the wholemeals of the 150 HEALTHGRAIN lines (Li et al. 2008) (Table 9).

More detailed information on the distribution of phytochemicals within the grain comes from analyses of individual tissues isolated by hand dissection and of bran, germ and flour fractions from milling. However, direct comparison of these datasets is difficult as different tissues/components have been analyzed in individual reports. Antoine et al. (2003, 2004) and Barron et al. (2007) reported the distributions of major PAs (*p*‐coumaric, sinapic and monomeric, dimeric and trimeric forms of ferulate) in hand‐dissected tissues. The latter study, which compared two cultivars, showed a mean total ferulate content of 1040 *μ*g/g dm in whole grain, 50 *μ*g/g dm in white flour, 500 *μ*g/g dm in the embryonic axis, 547 *μ*g/g dm in the intermediate layer, 3630 *μ*g/g dm in the scutellum, 6345 *μ*g/g dm in the outer pericarp, 8415 *μ*g/g dm in the aleurone and 10975 *μ*g/g dm in the hyaline layer. *p*‐Coumaric acid was not detected in white flour but was concentrated in the aleurone and, to a lesser extent, the outer grain layers with sinapic acid also being concentrated in the aleurone layer with only traces in white flour.

Chung and Ohm (2000) determined the contents and compositions of tocols in dissected wheat grain tissues, whereas Piironen et al. (1986) and Holasova (1999) reported analyses of milling fractions, with generally good agreement between the two approaches. The content of total tocols in wholegrain ranged from 40 to 58 *μ*g/g, with low contents in the purest white flour/starchy endosperm fractions (17–20 *μ*g/g) and higher contents in the bran layers (76–95 *μ*g/g) and germ (from hand dissection only) (181–320 *μ*g/g).

Chen and Geddes (1945) (quoted in Chung et al. 2009) reported that carotenoids are concentrated in the germ with a total concentration of 4.13–11.04 *μ*g/g compared with 0.88–2.22 *μ*g/g in bran and 1.57–2.18 *μ*g/g in the endosperm. Zhou and Yu (2005) have also recently reported the presence of *β*‐cryptoxanthin in wheat bran as well as *β*‐carotene, lutein, and zeaxanthin.

#### Health benefits of phytochemicals

Phenolic acids exhibit strong antioxidant activity and the total phenolic content is strongly correlated with total antioxidant activity (Adom et al. 2003; Beta et al. 2005). The relevance of antioxidant properties for human health is widely debated, with insufficient evidence being available to convince many health professionals or support health claims. However, there is increasing evidence that phenolic compounds, including ferulic acid (the major phenolic acid in wheat), improve vascular function in humans (Katz et al. 2001, 2004; Vauzour et al. 2010; Rodriguez‐Mateos et al. 2013) and animal models (Alam et al. 2013; Badawy et al. 2013; Suzuki et al. 2007). Whereas free (and possibly also conjugated) phenolic acids are absorbed in the small intestine, a high proportion of the total phenolic acids in wheat is ferulic acid, and to a lesser extent *p*‐coumaric acid, which are bound to arabinoxylan fiber. Bound ferulate, *p*‐coumarate and diferulate are all released by fermentation in the colon (Buchanan et al. 1996; Kroon et al. 1997; Andreasen et al. 2001) making them available for uptake (reviewed by Vitaglione et al. 2008). Ferulic and *p*‐coumaric acids have also been reported to have antiproliferative effects on human Caco‐2 colon cancer cells, but this effect has not been confirmed in vivo.

Studies have also shown activities of cereal alkylresorcinols (phenolic lipids) but only in cell and animal models (reviewed by Piironen et al. 2009).

Phytosterols and tocols both have established health benefits which do not apply specifically to wheat. Phytosterols are integral components of plant cell membranes and have well‐documented cholesterol‐lowering effects in humans with accepted health claims in Europe (EU Register on Nutrition and Health Claims, http://ec.europa.eu/nuhclaims/).

Although the name “vitamin E” is commonly applied to all tocols they differ in their biological activity with *α*‐tocopherol being the most active form. It is therefore usual to define their relative activities in International Units of *α*‐tocopherol equivalents, which are 1 mg per mg for *α*‐tocopherol and 0.5, 0.1, and 0.33 mg *α*‐tocopherol equivalents per mg for *β*,* γ,* and *δ*‐tocopherols, respectively. Of the tocotrienols only the *α* form has significant vitamin E activity, with an *α*‐tocopherol equivalent of 0.3 mg per mg. The biological activity of tocols is reviewed by Bramley et al. (2000) and Piironen et al. (2009).

### B vitamins

The B vitamin complex comprises eight water‐soluble components which often occur together in the same foods and were initially considered to be a single compound. Cereals are dietary sources of several B vitamins, particularly thiamine (B1), riboflavin (B2), niacin (B3), pyridoxine (B6), and folates (B9). There has been considerable debate on whether to introduce fortification of flour with folates (B9), on a voluntary or compulsory basis, with no internationally accepted policy (Lawrence et al. 2009; WHO, 2009).

All B vitamins are concentrated in the bran and/or germ (Piironen et al. 2009), with white flour containing significantly lower contents than wholemeal (Table 10). Variation in the contents of B vitamins has also been reported, particularly in the contents of thiamine (B1), riboflavin (B2), and niacin (B6) in white flour (Table 10).

**Table 10:** Variation in the content of B vitamins in wheat

Component	Number of lines	Range of content (*μ*g/g dw)	Fold variation	Mean content (*μ*g/g dw)	References
Wholegrain					
Folates (B9)	150	0.32–0.77	2.4	0.56	1
Thiamine (B1)	26	6.90–11.7	1.7	8.55	2
Thiamine (B1)	49	2.59–6.13	2.4	3.74	3
Riboflavin (B2)	26	0.86–1.07	1.2	0.96	2
Riboflavin (B2)	49	0.48–1.07	2.2	0.71	3
Niacin (B3)	26	0.69–1.18	1.7	0.87	2
Pyridoxine (B6)	26	1.6–2.2	1.4	1.91	2
Pyridoxine (B6)	49	1.44–3.16	2.2	2.2	3
White flour					
Folates (B9)	3	0.16–0.204	1.3	0.185	4
Thiamine (B1)	9	1.25–2.2	1.8	1.68	3
Thiamine (B1)	95	0.57–15.2	26.7	2.72	5
Riboflavin (B2)	9	0.43–0.58	1.3	0.49	3
Riboflavin (B2)	95	0.11–8.4	76.4	0.84	5
Niacin (B3)	95	6.84–100.3	14.7	21.51	5
Pyridoxine (B6)	9	0.27–0.52	1.93	0.43	3
Pyridoxine (B6)	16	0.074–0.349	4.7	0.209	6

References: 1. Piironen et al. 2008; 2. Shewry et al. 2011; 3. Batifoulier et al. 2006; 4. Gujska and Kuncewicz 2005; 5. Ranum et al. 1980; 6. Sampson et al. 1996.

Niacin (B3) is of particular concern as only a proportion of the total present in cereals is bioavailable. Niacin deficiency leads to pellagra, which was historically associated with consumption of diets based largely on maize flour. Carter and Carpenter (1982) reported that wheat bran contained only traces of free niacin, and that only 24% of the bound niacin was bioavailable. The bioavailability increased to 62% when the bound niacin was treated with alkali to convert it to free nicotinic acid. The bioavailable forms of niacin (the sum of nicotinamide and nicotinic acid after acid hydrolysis) were also determined by Shewry et al. (2011) on wholemeal flours of the HEALTHGRAIN winter wheat lines on four sites (UK, Poland, France and Hungary) in 2007. The bioavailable niacin content of these samples (0.16–1.74 *μ*g/g dw) was about 10–20% of the total niacin content reported in previous studies.

### Methyl donors (betaine, choline)

Although wheat is rarely discussed as a source of methyl donors (betaine and choline), it is one of the richest known dietary sources of betaine. Data from the HEALTHGRAIN project showed a threefold range in the content of betaine in wholemeal and a 1.55‐fold range in the content of choline (Corol et al. 2012) (Table 11).

**Table 11:** Variation in the contents of methyl donors (betaine and choline) in wholemeal of wheat

Component	Number of lines	Range of content	Fold variation	Mean content	References
Wholemeal					
Betaine	150	970–2940 *μ*g/g dm	3.03	1596 *μ*g/g dm	1, 2
Betaine	5	747–1503 *μ*g/g dw	2.01	1020 *μ*g/g dm	3
Choline	150	180–280 *μ*g/g dm	1.55	221 *μ*g/g dm	1, 2
Choline	5	76–135 *μ*g/g dw	1.78	110 *μ*g/g dm	3
White flour					
Betaine	6	166–326 *μ*g/g dw	1.96	239 *μ*g/g dm	3
Choline	6	54–65 *μ*g/g dw	1.21	58 *μ*g/g dm	3

References: 1. Shewry et al. 2013; 2. Corol et al. 2012; 3. Bruce et al. 2010.

Both compounds are concentrated in the bran fractions with low levels in white flour. Bruce et al. (2010) reported ranges from 165.9 to 325.8 *μ*g/g dw betaine and 53.6 to 64.6 *μ*g/g dw choline in six white wheat flours, compared with 747.0 to 1502.7 *μ*g/g dw betaine and 75.9 to 134.9 *μ*g/g dw choline in five wholegrain samples (Table 11).

Similarly, analyses of fractions from a single wheat line showed 8670 *μ*g/g dw betaine and 1020 *μ*g/g dw choline in bran, 15530 *μ*g/g dw betaine and 2090 *μ*g/g dw choline in aleurone and 230 *μ*g/g dw betaine and 280 *μ*g/g dw choline in white flour (Graham et al. 2009). Likes et al. (2007) showed that the contents of choline and betaine were 14.4 and 291.2 mg/100 g in wholemeal, 47.3 and 1293.3 mg/100 g in bran, 114.9 and 1163.5 mg/100 g in germ, and 3.6 and 71.8 mg/100 g in the purest white flour fraction.

#### Health benefits of methyl donors

High plasma homocysteine (hyperhomocysteinemia) is a major risk factor in CVD, with homocysteine produced by demethylation of methionine being removed either by remethylation to methionine, metabolism to give cysteine or conversion to S‐adenosylhomocysteine. The remethylation of homocysteine requires a methyl donor, either folate (vitamin B9) or betaine ((*N,N,N,*‐trimethyl) glycine) or choline (which can be converted to betaine in animals). Betaine and choline can also substitute for folate in other methylation reactions including the methylation of DNA (Zeisel and Blusztajn 1994; Niculescu and Zeisel 2002; Ueland et al. 2005).

Humans obtain betaine almost solely from their diet, but it can also be produced in humans by the irreversible conversion of choline. Wheat contains the highest reported levels of betaine of all plant foods, 12.9 and 15 mg/g in bran and 2.91 mg/g in wholegrain, with lower levels of free choline (about 0.5 mg/g in bran and 0.14 mg/g in wholegrain) (Zeisel et al. 2003; Likes et al. 2007). However, the content of betaine in 150 wheat lines varied by threefold (0.97–2.94 mg/g) and of choline from 0.18 to 0.28 mg/g (Corol et al. 2012).

A cross‐sectional analysis in 1477 women (part of the Nurses’ Health Study) showed that the total dietary intake of choline+betaine was inversely associated with total homocysteine in plasma (Chiuve et al. 2007), indicating the importance of adequate dietary intake. Intervention studies with aleurone‐rich foods (Price et al. 2010) and with wholegrain foods (Ross and Bruce 2011) have also shown that increased total intakes and plasma concentrations of betaine were associated with improvements in a number of biomarkers of health, including decreased total plasma homocysteine and LDL cholesterol. A more recent study has shown that the consumption of minimally processed wheat bran, and particularly the aleurone fraction, resulted in substantial postprandial increases in plasma betaine concentrations, including when aleurone fractions were incorporated into bread (Keaveney et al. 2015). EFSA have accepted a health claim relating to betaine, but this can only be applied to products containing at least 500 mg/portion (contributing to a daily intake of 1.5 g) (http://ec.europa.eu/nuhclaims/). This claim has not been applied to wheat products but it is likely even the lower betaine levels present in wheat products will contribute to reduced risk of CVD.

### Heritability of variation in wheat grain composition

It is clear that there is substantial variation in wheat grain composition, including the content and composition of DF and putative “bioactive” components. However, this variation can result from three effects: genetic differences between lines (genotype), environmental conditions (including the weather, soil conditions and agronomy) and interactions between the genotype and environment. Comparison of the compositions of sets of wheat lines allows the variation to be apportioned between these three effects, with a high contribution of the genotype meaning that the trait is highly heritable and hence the variation is available to breeders. We will therefore review our current knowledge of the genetic control and heritability of wheat grain composition.

**Figure 2:**
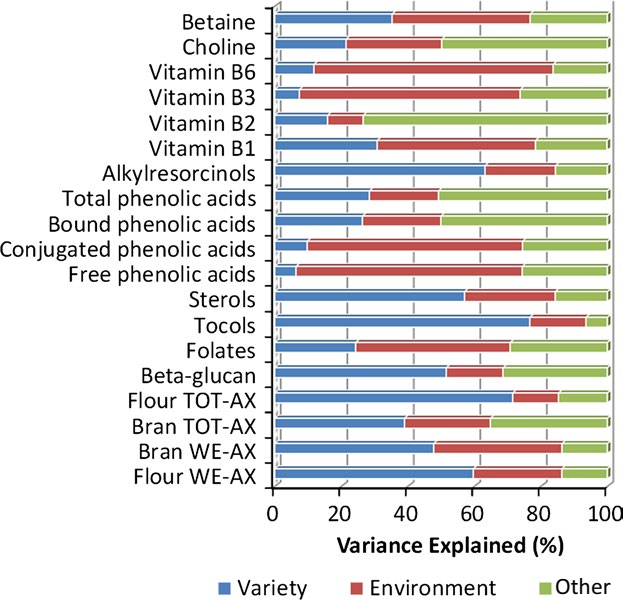
Summary of the heritability of dietary fiber and other components in wheat grain, based on the HEALTHGRAIN study. “Other” includes variance ascribed to Genotype × Environment interactions and/or error.

The “broad sense heritability” of grain components can be calculated by comparing the compositions of samples of multiple genotypes grown in multiple environments (sites and or years). Relatively little information is available on the heritability of most grain components, with the most complete series of studies being carried out under the HEALTHGRAIN program. This included multisite trials, in which 23–26 cultivars were grown in either four environments (for B vitamins) or six environments (for other components) (Shewry et al. 2010, 2011; Corol et al. 2012). The results of these studies are summarized in Fig. 2. Alkylresorcinols, sterols and tocols all show high heritability (above 50%), whereas B vitamins, methyl donors (choline, betaine), and phenolic acids all have low heritability with high effects of environment (or G × E interactions).

DF components show high heritability, in both white flour and bran fractions (data for wholemeal are not available). In particular, TOT‐AX and WE‐AX in white flour show heritabilities of about 70% and 60%, respectively.

Similar high heritabilities for AX have been shown in several other reported studies. Hong et al. (1989) analyzed 18 wheat lines (seven hard red winter, seven hard white winter and four club wheats) grown on two sites in Washington State, USA, and calculated that the genotypic variance for water‐soluble pentosans (i.e., WE‐AX) in wholemeal was 1.6 times the environmental variance and for total pentosans (i.e., TOT‐AX) 2.4 times. Martinant et al. (1999) reported broad sense heritabilities of 0.75 for WE‐AX of flour and 0.80 for the viscosity of aqueous extracts of flour (which is largely determined by WE‐AX) from 19 cultivars grown on three locations in France, whereas Dornez et al. (2008) reported broad sense heritabilities of 0.53 for TOT‐AX and 0.96 for WE‐AX in wholemeals of 14 cultivars grown in Belgium for 3 years. Similar studies of wholemeal samples of five durum wheat cultivars grown under four agronomic regimes gave genotype/environment ratios of 4.5 for TOT‐AX and 4.9 for WE‐AX (Lempereur et al. 1997). High heritability of WE‐AX and TOT‐AX in flour was also reported by Finnie et al. (2006) who analyzed seven spring wheat lines grown in 10 environments and 20 winter wheat lines grown in 12 environments.

However, Li et al. (2009) reported contrasting results for wholemeals of 25 hard winter wheats and 25 hard spring wheats grown at three locations. They showed that environment had a much greater effect than genotype on WE‐AX and TOT‐AX in the winter lines, by more than an order of magnitude, and a greater impact than genotype on WE‐AX (but not TOT‐AX) in spring wheats. Hence, the relative effects of G and E depended on the genotypes and environments.

## Conclusions

The consumption of wheat is increasing globally, including in countries with climates that are not suitable for wheat production. Wheat‐based foods provide a range of essential and beneficial components to the human diet, including protein, B vitamins, DF, and phytochemicals. These components may also vary widely in amount and composition due to effects of genotype and environment. DF is particularly important as consumption is associated with reduced risk of CVD, type 2 diabetes, and certain forms of cancer. DF components also have high heritability and their amount should therefore be amenable to manipulation by breeding, particularly if molecular markers can be established to reduce the need for expensive chemical analyses during screening.

## Conflict of Interest

None declared.
